# Seroprevalence and risk factors of *Toxoplasma gondii* infection in children with leukemia in Shandong Province, Eastern China: a case—control prospective study

**DOI:** 10.7717/peerj.6604

**Published:** 2019-03-13

**Authors:** Na Zhou, Haiyang Fu, Zhongjun Wang, Hailei Shi, Yang Yu, Tingting Qu, Longlong Wang, Xiangyan Zhang, Lin Wang

**Affiliations:** 1 Department of Pediatrics, The Affiliated Hospital of Qingdao University, Qingdao, Shandong, China; 2 Department of Pathology, Affiliated Hospital of Qingdao University, Qingdao, China; 3 Department of Clinical Laboratory, The Affiliated Hospital of Qingdao University, Qingdao, China; 4 Department of Urinary Surgery, The Affiliated Hospital of Qingdao University, Qingdao, China

**Keywords:** *Toxoplasma gondii*, Risk factors, Leukemia children, Seroprevalence

## Abstract

Limited information is available concerning the epidemiology of *Toxoplasma gondii* infection in children with leukemia in Eastern China. Therefore, a case-control study was conducted to estimate the seroprevalence of toxoplasmosis in this patient group and to identify risk factors and possible routes of infection. Serum samples were collected from 339 children with leukemia and 339 age matched health control subjects in Qingdao from September 2014 to March 2018. Enzyme linked immunoassays were used to screen anti- *T. gondii* IgG and anti- *T. gondii* IgM antibodies. Forty-eight (14.2%) children with leukemia and 31 (9.1%) control subjects were positive for anti-*T. gondii* IgG antibodies (*P* < 0.05), while 13 (3.8%) patients and 14 (4.1%) controls were positive for anti-*T. gondii* IgM antibodies (*P* = 0.84). Multivariate analysis showed exposure to soil and a history of blood transfusion were risk factors for *T. gondii* infection. Compared with IgG, patients with a history of blood transfusion were more likely to present anti- *T. gondii* IgM (*P* = 0.003). Moreover, patients with chronic lymphocytic leukemia and acute lymphocytic leukemia had higher *T. gondii* seroprevalence in comparison to control subjects (*P* = 0.002 and *P* = 0.016, respectively). The results indicated that the seroprevalence of *T. gondii* infection in children with leukemia is higher than that of healthy children in Eastern China. This information may be used to guide future research and clinical management, and further studies are necessary to elucidate the role of *T. gondii* in children with leukemia.

## Introduction

Leukemia is one of the most common malignant tumors in children, with more than 8,000 new cases and 4,000 disease-related deaths in China in 2013 (Chen et al., 2017). The morbidity has increased steadily due to changes in lifestyle in recent years. Complications of infection are one of the most severe risk factors that lead to death for leukemia patients. There have been comprehensive surveys for infections conducted in patients with malignancy including bacteria, virus and fungus (Mori et al., 2014; Vehreschild et al., 2014; Wang et al., 2017), but parasites, especially opportunistic infection parasites, in patients who are immunocompromised by virtue of neoplastic disease have received relatively little attention (Cong et al., 2015; Zueter et al., 2014).

One of the most frequent protozoan parasites causing opportunistic pathogenic infection in immunocompromised hosts is *Toxoplasma gondii*, which is an intracellular protozoan that chronically infects approximately a third of the world’s population (Montoya & Liesenfeld, 2004). Human infection results from ingestion of oocysts from insanitary food or water containing *T. gondii* tissue cysts (The Lancet Infectious, 2012). In individuals with normal immunity, acute acquired *T. gondii* infection is generally self-limiting and asymptomatic due to efficient immunity that limits the propagation of the multiplying tachyzoite stage. During this period, the tissue cysts are contained by the humoral and cellular immune system, including macrophages and T-lymphocytes (Montoya & Liesenfeld, 2004; Shaw et al., 2009). However, *T. gondii* still holds viable in the form of tissue cysts throughout the whole life of its host (Montoya & Liesenfeld, 2004). In immunocompromised individuals, such as patients with malignant tumors or collagen tissue disease, or transplant recipients under immunosuppressive therapy, cysts have greater propensity to relapse and disseminate, which can cause serious clinical consequences and even death (Donahoe et al., 2017; McLeod et al., 2009; Nimir et al., 2010; Syrogiannopoulos et al., 2002).

The treatment methods for leukemia are chemotherapy, radiotherapy, immunotherapy and combination therapy. These managements may cause immune system dysfunction, which predisposes the patient to the development of toxoplasmosis. Moreover, *T. gondii* infections are often ignored in the process of clinical diagnosis and treatments. Therefore, knowing the factors that increase susceptibility to *T. gondii* and recognising the early symptoms of *T. gondii* infection in patients with leukemia will promote the prevention of *T. gondii* infection and ideally increase the ability to assess patients’ needs. Additionally, several questions remain unclear: first, the prevalence of *T. gondii* infection in children with leukemia in Eastern China is still unknown; second, it is unclear whether demographic or lifestyle variables increase the risk of *T. gondii* infection in children with leukemia. Thus, we conducted this study to investigate the risk of toxoplasmosis in children with leukemia and the association between *T. gondii* infection and different risk factors.

## Methods

### Subjects

During September 2014 to March 2018, serum samples were collected from 339 primary children with leukemia who presented to the Affiliated Hospital of Qingdao University for diagnosis and treatment. No patients had received IVIG therapy and/or immunotherapy before blood collection. The ages of the children with malignancy were 0–14 years old. For control subjects, 339 children who participated in health screenings at the hospitals were recruited, matched with children with malignancy by age, gender, and residence. The study was approved by the Ethics Committee of the Affiliated Hospital of Qingdao University (No. 20141349) and all patients/guardians had signed informed consent.

### Sample collection

Approximately 2 mL of venous blood were drawn from participants who gave their consent to participate in this study. Blood samples were left for 2 h at room temperature to allow clotting, and were centrifuged at 3,000 rpm for 10 min. The sera were collected in EP tubes and stored at −80 °C until tested.

### Socio-demographic and clinical data

Socio-demographic data including age, sex, residence and parent’s occupation were obtained from all participants. Behavioral data included any history of contact with stray cats, contact with pigs, any cats or dogs kept at home, consumption of raw/undercooked meat, consumption of raw vegetables and fruits, exposure to soil, and source of drinking water (Cong et al., 2015). Clinical data collected in patients included the histological type of leukemia, and history of blood transfusion and chemotherapy. Data were obtained from the patients/guardians and medical records, and patients/guardians were blinded to infectious status before data were obtained.

### Serological assay

Sera were tested for the presence of IgG and IgM antibodies against *T. gondii* using commercially available enzyme immunoassay kits (ELISA) (Demeditec Diagnostics GmbH, Germany). This procedure was performed according to the manufacturer’s instructions. Positive and negative serum controls were included in every plate. Samples from children with leukemia and control subjects were randomly mixed.

### Statistics

Statistical analysis was performed using the statistical software SPSS 19.0. Chi-square test or Fisher exact test were used to analyze the anti-*T. gondii* antibody seroprevalence in socio-demographic data. Multivariate logistic regression models were used to adjust for potential confounders. Variables associated with *T. gondii* infection in univariate analysis (*P* ≤ 0.25) were included in a multivariate logistic regression analysis. Adjusted odds ratio (OR) and 95% confidence interval (CI) were calculated by multivariate analysis to identify independent risk factors for *T. gondii* infection. Results with *P*-value <0.05 were considered statistically significant.

## Results

### Epidemiology and risk factors for children with leukemia with *T. gondii* infection

A total of 678 individuals (339 children with leukemia and 339 health controls) were tested between September 2014 and March 2018. Fifty-seven and 33 (16.8% vs 9.7%, *P* < 0.05) samples were positive for anti-*T. gondii* antibodies in children with leukemia and control subjects, respectively. A significant difference (*P* < 0.05) was detected in the seroprevalence of anti-*T. gondii* IgG antibodies in 48 children with leukemia (14.2%) versus 31 control subjects (9.1%). Thirteen patients tested positive for *T. gondii* IgM antibodies (3.8%), compared with 14 controls (4.1%) (*P* = 0.84). The details of the children with leukemia and control subjects, including age distribution, gender, resident area, parent’s occupation and data of lifestyle and clinical treatment, are shown in Table 1. Our analysis revealed significant differences between the 6∼9-year-old age group (8/81, 9.9%) and the ≤ 2-year-old age group (22/101, 21.8%) (*P* = 0.031). *T. gondii* seroprevalence was higher in male leukemia patients (38/188, 20.2%) than in female patients (19/151, 12.6%), but there was no statistical difference (*P* = 0.06).

Univariate analysis showed some lifestyle and clinical variables where *P* ≤ 0.25, including contact with swine, exposure to soil, and history of transfusion and chemotherapy. In multivariate analysis, only exposure to soil (OR, 1.82; 95% CI [0.92–2.89]; *P* = 0.046) and history of transfusion (OR, 2.27; 95% CI [1.27–4.08]; *P* = 0.006) were significantly associated with *T. gondii* infection in leukemia patients (Table 2). Additionally, we analyzed the different results between IgG and IgM against *T. gondii* in children with leukemia who received blood transfusions. Compared with IgG, patients with blood transfusion were more likely to present anti- *T. gondii* IgM (Table 3). No significant associations between *T. gondii* status and other variables were found in the present study.

### Seroprevalence among children with leukemia

The seroprevalence of different histological types of leukemia is presented in Table 4. The highest seroprevalence of *T. gondii* was detected in patients with chronic lymphocytic leukemia (CLL) (28.9%), followed by acute lymphocytic leukemia (ALL) (18.1%), acute myeloblastic leukemia (AML) (15.9%), and chronic myeloblastic leukemia (CML) (7.5%). Compared with control subjects, patients with CLL and ALL had significantly higher seroprevalence (*P* = 0.002 and *P* = 0.016, respectively).

## Discussion

*Toxoplasma gondii* infection is increasingly being reported among patients with leukemia, and the higher seroprevalence of anti-*T. gondii* antibodies among leukemia patients reflects latent association between *T. gondii* infection and leukemia (Gharavi, Roozbehani & Mandeh, 2017; Huang et al., 2016; Yazar et al., 2004); however, both the epidemiology and risk factors for this are unclear. Moreover, information about *T. gondii* infection in children with leukemia is also limited. Therefore, we assessed the seroprevalence of anti-*T.gondii* antibodies in 339 children with leukemia from Shandong province, Eastern China, and explored the risk factors of *T. gondii* infection in children with leukemia.

**Table 1:** Seroprevalence of *T. gondii* infection in children with leukemia and control subjects in Eastern China.

		Leukemia Children (*N* = 339)			Controls (*N* = 339)			
	Prevalence of *T. gondii* infection				Prevalence of *T. gondii* infection			
Characteristic	No. tested	No. positive	%	P	No. tested	No. positive	%	P
Age (years)								
≤2	101	22	21.8	Reference	98	6	6.1	Reference
3∼6	101	19	18.8	0.6	119	12	10.1	0.29
7∼10	81	8	9.9	0.031	66	11	16.7	0.03
11∼14	56	8	14.3	0.15	56	4	7.1	0.81
Gender								
Male	188	38	20.2	0.06	180	17	9.4	0.85
Female	151	19	12.6		159	16	10.1	
Residence area								
Urban	182	30	16.5	0.29	254	25	9.8	0.91
Rural	157	27	17.2		85	8	9.4	
Contact with cats								
Yes	75	14	18.7	0.63	69	9	13.1	0.29
No	264	43	16.3		270	24	8.9	
Contact with dogs								
Yes	69	10	14.5	0.56	47	2	4.3	0.28
No	270	47	17.4		292	31	10.6	
Contact with swine								
Yes	112	15	13.4	0.24	59	3	5.1	0.19
No	227	42	18.5		280	30	10.7	
Consumption of raw/ undercooked meat								
Yes	36	6	16.7	0.98	83	8	9.6	0.97
No	303	51	16.8		256	25	9.8	
Consumption of raw vegetables								
Yes	84	16	19.1	0.53	212	19	9	0.54
No	255	41	16.1		127	14	11	
Exposure to soil								
Yes	95	23	24.2	0.04	58	4	6.9	0.42
No	244	34	13.9		281	29	10.3	
Source of drinking water								
Well + river	30	4	13.3	0.59	29	3	10.3	0.75
Tap	309	53	17.2		310	30	9.7	
Parent’s occupation								
Farmer	105	21	20	0.29	119	14	11.8	0.35
Worker	234	36	15.4		220	19	8.6	
Surgery history								
Yes	75	14	18.7	0.63				
No	264	43	16.3					
Blood transfusion history								
Yes	102	26	25.5	0.005				
No	237	31	13.1					
Chemotherapy history								
Yes	236	36	15.3	0.25				
No	103	21	20.4					

Our results demonstrated that children with leukemia were more likely to be infected with *T. gondii* when compared with healthy controls (16.8% vs 9.7%, *P* = 0.002), though there was no significant difference between children with leukemia and controls with regard to the level of IgM antibodies, which was consistent with previous studies (Gharavi, Roozbehani & Mandeh, 2017). In healthy children, *T. gondii* infection rate may vary by age, and older children may have higher propensity for *T. gondii* infection (Fan et al., 2012; Fu et al., 2014; Marchioro et al., 2015), but in this present study, children with leukemia ≤ 2-years-old had the highest seroprevalence than any other subgroup. *T. gondii* is an opportunistic pathogen—in healthy individuals, *T. gondii* infection rate may increase with age because there is more possibility of *T. gondii* infection with time (Meng et al., 2015). However, the mechanisms by which younger leukemia patients are inclined to develop toxoplasmosis have not been comprehensively explained. One speculation for this is that patients with leukemia are immunocompromised and young patients may have immunities too frail to control opportunistic infections (Cong et al., 2015). This suggests that younger leukemia patients might be more susceptible to *T. gondii* infection, and further studies based on larger and well-distributed sample sizes are needed to confirm this conclusion.

**Table 2:** Multivariate analysis of selected characteristics of leukemia patients and their association with *T. gondii* infection.

Characteristic^a^	Adjusted odds ratio^b^	95% CI	*P*
Gender	1.43	1.08–2.22	0.09
Contact with swine	1.21	0.64–1.71	0.21
Exposure to soil	1.82	0.92–2.89	0.046
Blood transfusion history	2.27	1.27–4.08	0.006

**Notes.**

a The variables included were those with a *P* < 0.25 obtained in the univariate analysis.

b Adjusted by age.

**Table 3:** Seroprevalence of *T. gondii* antibodies in children with leukemia with history blood transfusion.

	Blood transfusion history (No.)		*P*
	Yes	No	
Anti- *T. gondii* IgG antibodies				
No. Positive (%)	19(18.6)	29(12.2)	0.122^a^
No. Negative (%)	83(81.4)	208(87.8)	
Anti- *T. gondii* IgM antibodies				
No. Positive (%)	9(8.8)	4(1.7)	0.003^b^
No. Negative (%)	93(91.2)	233(98.3)	

**Notes.**

a Chi-square tests.

b Fisher exact test were used.

**Table 4:** Clinical diagnosis and seroprevalence of *T. gondii* in children with leukemia in Eastern China.

Clinical diagnosis	No. tested	No. positive	%	*P*
Acute myeloblastic leukemia	132	21	15.9	0.06
Acute lymphoblastic leukemia	116	21	18.1	0.016
Chronic myeloblastic leukemia	53	4	7.5	0.61
Chronic lymphocytic leukemia	38	11	28.9	0.002

**Notes.**

As compared with 9.7% seroprevalence of anti- *T. gondii* antibodies in controls (33/339).

Previous reports indicated that contact with pigs and cats, consumption of raw/undercooked meat and exposure to soil were risk factors for *T. gondii* infection in cancer patients (Alvaradoesquivel et al., 2010; Cong et al., 2015; Zhou et al., 2018). In the present study, multivariate analysis showed that in leukemia patients, *T. gondii* seroprevalence was not associated with any lifestyle and feeding habits, except exposure to soil. As the definitive hosts of *T. gondii*, felids play a vital role in the transmission of this parasite (Webster, 2010). Cats infected with *T. gondii* shed millions of oocysts that can survive in soil for many years, and a single oocyst has the potential to cause full-blown infection and toxoplasmosis if ingested by humans (Tian et al., 2017a; Webster, 2010). Moreover, contact with cats and drinking water contaminated with cat feces also increases the risk of *T. gondii* infection (Tian et al., 2017a; Tian et al., 2017b). Although humans can be infected with *T. gondii* through exposure to soil contaminated with *T. gondii* oocysts, little attention has been paid to their role. Therefore, there is an urgent need to educate the general public and medical professionals about this risk factor of *T. gondii* infection in immunocompromised individuals, particularly leukemia patients, in order to reduce the risk of toxoplasmosis.

In our study, patients with a history of blood transfusion had a significantly higher seroprevalence than control subjects, which is a similar result to a study conducted by Alvarado-Esquivel et al. (2018). Moreover, compared with IgG, patients with blood transfusions were more likely to present anti- T. gondii IgM. Previous studies have demonstrated that intracellular tachyzoites can disseminate into all organs through blood circulation (Harker, Ueno & Lodoen, 2015), and hosts might be infected with T. gondii by infusing blood infected with tachyzoites (Alvarado-Esquivel et al., 2018). Positive T. gondii IgM is often defined as an acute infection, but isolated IgM without an IgG positive is interpreted as a chronic infection or false positive (Reshika et al., 2015). However, anti-T. gondii IgG seropositivity appears early after infection and high levels of specific anti-T. gondii IgG antibodies can be present in patients with recent infections (Liesenfeld et al., 2001). In this study, 11 samples (nine children with leukemia and two healthy controls) were detected with isolated positive IgM, and all of them were diagnosed with toxoplasmosis after a few days by clinicians according to clinical features. Chemotherapy is frequently used for the treatment of leukemia, and most children with leukemia who received transfusion were immunocompromised and struggled to produce enough immunoglobulin (e.g., A and G) to resist parasite infections. Moreover, microbiotics (such as amoxicillin, azithromycin and sulfamethoxazole) were also used for patients to prevent against pathogen infection. Sulfamethoxazole might contribute in preventing toxoplasmosis and turning positive antibodies into negative ones, so, it is within reason that there are significant differences in IgM, rather than in IgG antibodies, for patients with a history of transfusion.

Among the various histological types of leukemia, seroprevalence of CLL and ALL were significantly higher than controls. The progression of apoptosis and anti-apoptosis was regulated by miRNAs (He et al., 2015). *Toxoplasma gondii* can phagocytose these miRNAs, which might result in the hosts’ gene expression irregularity, and can cause carcinogenesis (Gharavi, Roozbehani & Mandeh, 2017; Saçar, Bağcı& Allmer, 2014). Nevertheless, malignant tumors were associated with defects in cell-mediated immunity, which was defined as a lymphocytic exhaustion phenomenon. When strengthened by treatment with defects in cell-mediated immunity, the patients struggled to resist intracellular pathogen infections and inclined toward development of toxoplasmosis (Cong et al., 2015; Yazar et al., 2004). This can partially explain the higher seroprevalence in lymphocytic leukemia than in granulocytic leukemia.

*Toxoplasma gondii* infections are often ignored in the process of clinical diagnosis and treatment, which may result in severe toxoplasmosis. Although further studies should be conducted to explore the causation between *T. gondii* infection and blood transfusion in leukemia patients, it is a pressing matter of the moment to encourage medical professionals to pay attention to the important role that exposure to soil and blood transfusion play in transmitting *T. gondii* infection to leukemia patients, and to test *T. gondii* routinely before donor blood is infused to patients. Moreover, a recent study conducted by Quinn et al. (2018) suggested that pentamidine effectively prevents Pneumocystis jirovecii pneumonia in pediatric oncology patients receiving immunosuppressive chemotherapy. Children with leukemia are immunosuppressive and struggle to resist parasite infections. Therefore, patients with leukemia should be monitored for early signs of toxoplasmosis and secondary prevention against *T. gondii* using trimethoprim or sufamethoxazole is necessary for patients with leukemia.

Additionally, the limitations of our results should be considered: first, the sample size was relatively small, rendering some findings inconclusive; second, the donors’ sera antibody statuses were unclear, so the positives for *T. gondii* antibody caused by donor derived antibody could not be excluded; third, we did not collect the clinical information on the treatment history of study participants, therefore, the effect of immunosuppressive and anti- pathogen therapies for antibody seroprevalence in children with leukemia was uncertain.

## Conclusion

In this study, we reported serological evidence of an association between *T. gondii* infection and children with leukemia. Exposure to soil and blood transfusion history were identified as risk factors for *T. gondii* infection. This information may be used to guide future research and clinical management. However, further studies are necessary to elucidate the role of *T. gondii* in children with leukemia.

##  Supplemental Information

10.7717/peerj.6604/supp-1 Supplemental Information 1 Socio-democlgraphic data of 339 children with leukemia and 339 health subjects were included

## Additional Information and Declarations

### Data Availability

The following information was supplied regarding data availability: Socio-demographic data of 339 children with leukemia and 339 healthy subjects are available as a Supplemental File.
